# Prevalence and animal level risk factors associated with *Trypanosoma evansi* infection in dromedary camels

**DOI:** 10.1038/s41598-022-12817-x

**Published:** 2022-05-27

**Authors:** Abdelfattah Selim, Hayat Ali Alafari, Kotb Attia, Muneera D. F. AlKahtani, Fatima M. Albohairy, Ibrahim Elsohaby

**Affiliations:** 1 Department of Animal Medicine (Infectious Diseases), Faculty of Veterinary Medicine, Benha University, Moshtohor-Toukh, Kalyobiya, 13736 Egypt; 2 Center of Excellence in Biotechnology Research, King Saud University, P.O. Box 2455, Riyadh, 11451 Saudi Arabia; 3 Department of Biology, College of Science, Princess Nourah bint Abdulrahman University, P.O. Box 84428, Riyadh, 11671 Saudi Arabia; 4 Extramural Research Department, Health Sciences Research Center, Princess Nourah bint Abdulrahman University, P.O. Box 84428, Riyadh, 11671 Saudi Arabia; 5 Department of Animal Medicine, Faculty of Veterinary Medicine, Zagazig University, Zagazig, 44511 Sharkia Egypt; 6 Department of Infectious Diseases and Public Health, Jockey Club of Veterinary Medicine and Life Sciences, City University of Hong Kong, Kowloon, Hong Kong

**Keywords:** Risk factors, Parasitology

## Abstract

Surra is a non-cyclic parasitic disease caused by *Trypanosoma evansi* (*T. evansi*) and spread by biting flies. The disease has a severe impact on camel health, productivity, and market value, posing a significant threat to food safety and the economy. In a cross-sectional study, 370 blood samples were collected from camels in three Egyptian governorates. Samples were tested using parasitological (thin blood smear (TBS)), card agglutination test for *T. evansi* (CATT), and PCR to estimate the prevalence of *T. evansi* infection*.* Overall, the prevalence of *T. evansi* among examined camels was 17.3%, 18.9% and 22.7% using TBS, CATT and PCR methods, respectively. The risk of *T. evansi* infection in older camels (> 10 years) is higher than that in young ones (odds ratio (OR) = 9; 95% CI: 3.5–23.1), particularly during spring (OR = 2.5; 95% CI: 1.1–5.7). Furthermore, females and poor conditioned camels were 2.6 and four times more likely to get infection than males and good conditioned camels, respectively. The level of agreement between diagnostics tests were perfect kappa (> 0.83). Moreover, CATT showed higher sensitivity (0.83; 95% CI: 0.74–0.91) than TBS (0.76; 95% CI: 0.66–0.85) and both had perfect specificity (100%). In conclusion, our findings revealed a high rate of *T. evansi* infection in camels from the three Egyptian governorates. The CATT is a good test for routine use in control program of trypanosomiasis in camels.

## Introduction

Trypanosomiasis is a vector-borne disease that affects both animals and human health in tropical and subtropical countries including Egypt, causing significant economic losses^1,2^. Camel trypanosomiasis “Surra” caused by *Trypanosoma evansi* (*T. evansi*) which is a member of the *Trypanosomatidae* family, genus *Trypanosome*, and subgenus *Trypanozoon*^3^. Mechanical transmission of the disease occurs by the biting of flies such as *Stomoxys*, *Tabanids,* and *Hippoboscids*^4^. The disease course ranged from acute infection with high mortalities to chronic infection with reduction in body weight, anemia, infertility and due to *T. evansi*'s immunosuppressive impact, it's generally accompanied with secondary infection. which makes clinical identification more difficult^5,6^.

In the absence of disease pathognomonic signs, a laboratory diagnosis is required to confirm infection. Direct microscopic examination of stained or wet blood films is used for parasitological identification, although it has a low sensitivity since parasitaemia is intermittent^7^. Additionally, the World Organization for Animal Health has suggested the card agglutination test for *T. evansi* (CATT/*T. evansi*) as a quick diagnostic test^8^. Since the introduction of molecular diagnostic techniques, several diagnostic assays based on trypanosomal DNA PCR detection have been developed. In a variety of hosts, PCR has been demonstrated higher sensitivity than standard parasitological approaches, with the added benefit of being able to identify parasites down to the subspecies level^9,10^. Moreover, *T. evansi* can be detected using a variety of target sequences, including ribosomal DNA, kinetoplast DNA, the internal transcribed spacer area, and VSG genes^11,12^.

To date, several studies were conducted in different areas of Egypt for detection and diagnosis of Trypanosomiasis in camel. In the northern west of Egypt, Sobhy, et al.^13^ found 20.6% of camels harboured *T. evansi* by staining blood films, and 64.3% were positive by PCR assay, while Barghash et al.^14^ and El-Naga & Barghash^15^ in the same area discovered trypanosomiasis was prevalent in camels with 20.9%, 65.9%, and 20.24%, 67.06% based on blood film and PCR assay, respectively. Although, several diagnostic techniques are available for determining the degree of the disease's prevalence, and morbidity in the field, it is currently difficult to estimate the disease's impact on camels in Egypt and the resulting economic loss. Therefore, the present study aimed to (1) assess the prevalence and risk factors associated with camel trypanosomiasis in three Egyptian governorates; (2) determine the diagnostic test characteristics of parasitological examination (thin blood smear (TBS)) and CATT for detection camel trypanosomiasis against PCR; and (3) estimate the agreement between different diagnostic tests for detection of *T. evansi* infection in dromedary camels in Egypt.

## Materials and methods

### Ethical statement

The study was conducted in accordance with Benha University's Declaration and was approved by the Faculty of Veterinary Medicine's Ethics Committee (protocol no.: BUFVTM 23-2-2022). The research was carried out in accordance with the ARRIVE criteria.

### Study area and sample size

**Figure 1:**
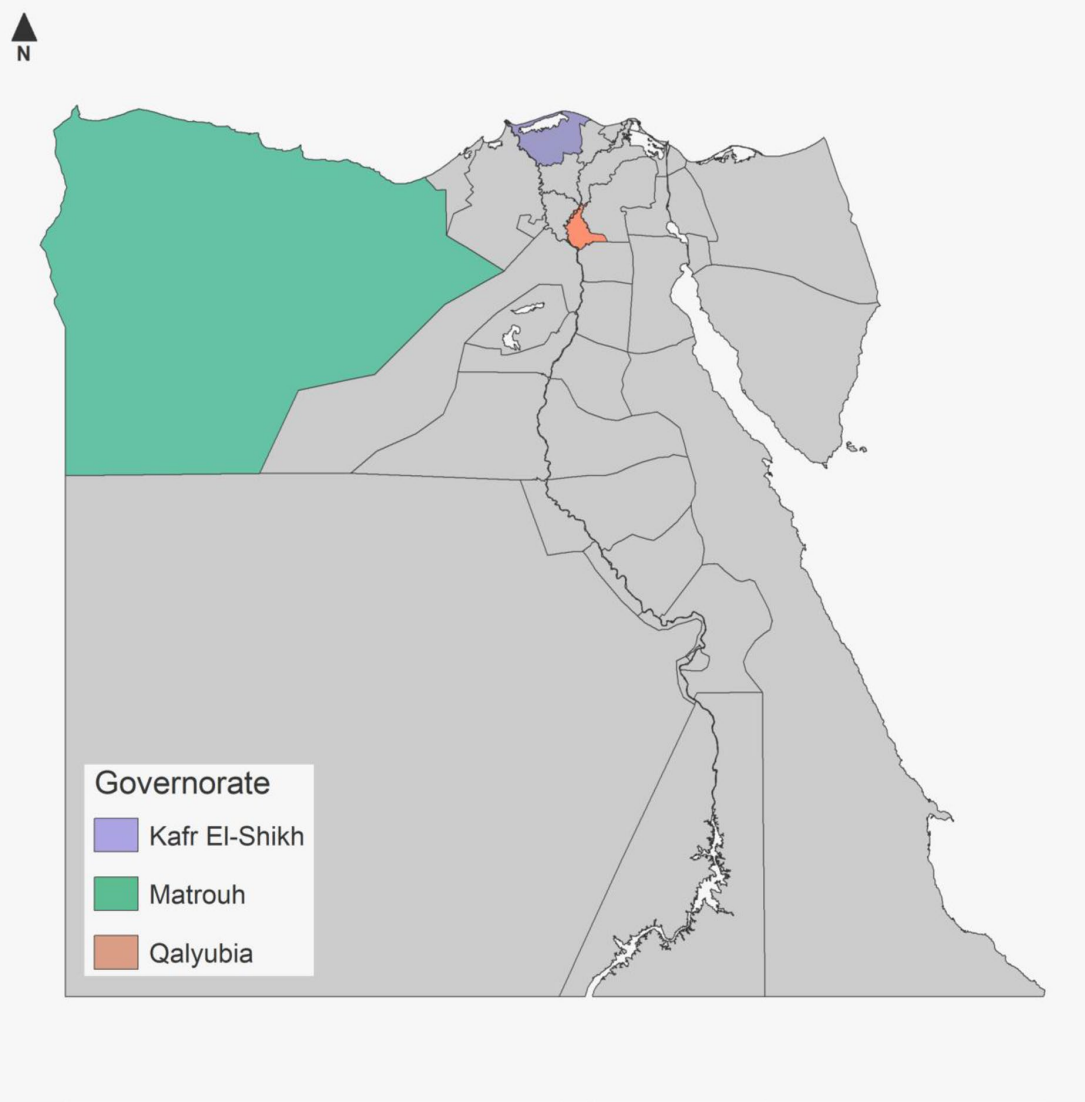
Map of Egypt showing the three sampled governorates.

Three Egyptian governorates namely; Qalyubia (30.41° N 31.21° E), Kafr ElSheikh (31°06′42″ N 30°56′45″ E) and Marsa Matrouh (31°20′ N 27°13′ E) which geographically located nearly from Mediterranean sea at the Northern Egypt were involved in the present study (Fig. 1). These districts have high camel population, which usually used as food source, also for drought and breeding. The climate of the selected areas called desert climate according to classification of Köppen-Geiger climate. The average annual temperature in these locations is 20 °C, while the average annual rainfall is 63 mm.

A cress-sectional study was carried out on *T. evansi* infection in dromedary camels during 2020 using a random sampling approach. The sample size was calculated according to Thrusfield formula^16^ using an expected seroprevalence of 4.7%^17^, a confidence level of 95% and desired precision of 5%:

$$n=\frac{{1.96}^{2} {P}_{exp } (1- {P}_{exp})}{{d}^{2}}$$

where *n* is the required sample size, *P*_*exp*_ is the expected prevalence and *d* is the desired precision.

The sample size required for this study was calculated to be 70 camels; however, to improve the precision of diagnostic test estimates, the sample size was extended to 370 camels^18^.

### Sampling and data collection

Three millilitres of blood were taken from jugular vein into tubes containing no anticoagulant and kept at room temperature until clot response was noticed. Then, the sera were separated by centrifugation at 1500×*g* for five minutes and kept at −20 °C until serological analysis. Additionally, three mL of blood were placed in tube with anticoagulant (EDTA) for parasitological and molecular examinations.

During animal sampling, data such as age, sex, season, body condition score (BCS) were collected. All participants provided informed verbal/written consent to participate in the study.

### Diagnostic tests

#### Parasitological examination

Thin blood smear (TBS) was prepared following standard procedures and stained by Giemsa stain for detection of *Trypanosoma* spp. under oil immersion objectives^19^. After looking at least 50 fields, the findings were decided.

#### Card agglutination test for trypanosomosis

The Card Agglutination Test for *T. evansi* (CATT/*T. evansi*) (Institute of Tropical Medicine, Antwerp, Belgium) was used to detect antibodies against *T. evansi* in sera of examined camels^20^, as directed by the manufacturer.

#### PCR

Using a commercially available kit (QIAamp DNA Blood Mini Kit, Qiagen), genomic DNA was isolated from 200 μL of whole camel blood and kept at −80 °C until further use. The extracted DNA was examined by PCR assays based on specific pair of primers for *T. evansi* targeting ITS1 rDNA gene, which previously evaluated by Zangooie, et al.^10^. A total reaction volume of 25 μL was used for the PCR amplifications, containing 1 μL of 10 pM primers, 12.5 μL of DreamTaq Green PCR Master Mix (2×) (Thermo Fisher, Germany), 5.5 μL of RNase free water, and 5 μL of DNA template.

PCR amplifications carried out in thermocycler (BioRad, USA). An initial denaturation stage at 94 °C for 30 s was followed by 35 cycles of 94 °C for 30 s, 58 °C for 30 s, 72 °C for 1 min, with a final extension step at 72 °C for 5 min and chilling at 4 °C. In all PCR runs, distilled water and (*T. evansi*-DNA) served as negative and positive controls, respectively. The amplified PCR products were detected on 2% agarose gel which stained by ethidium bromide.

### Statistical analysis

For descriptive and statistical data analysis, epidemiological data and diagnostic test results were entered into Stata Statistical Software v. 15 (Stata Corp, College Station, TX, USA). The proportions of camel trypanosomiasis were estimated as the ratio of positive camels to the total number of camels examined with the exact binomial confidence interval of 95% (95% CI). McNemar's test was used to compare the proportion of positive test results from two different tests^21^. The univariable association between risk factors potentially associated camel trypanosomiasis was investigated and a multivariable model was then built using a backward-elimination procedure with a *P*-value < 0.05 to retain variables. Hosmer–Lemeshow Goodness of fit statistics was used to assess the final model's fitness^22^.

Using PCR results as reference test, the applicability of TBS and CATT for the detection of camel trypanosomiasis, epidemiological diagnostic test characteristics (Se, Sp, predictive values, and accuracy) were calculated. The Se was defined as the proportion of camels infected with *T. evansi*, as determined by PCR, that were classified as positive by TBS/CATT. Conversely, Sp was defined as the proportion of non-infected camels that were classified as negative by TBS/CATT. Accuracy was defined as the proportion of camels that were correctly classified as infected and non-infected by TBS/CATT. The Cohen's kappa statistic was used to assess the overall agreement between two tests. The prevalence-adjusted and bias-adjusted kappa (PABAK) statistic was calculated to account for the bias introduced by the disease's prevalence and the tendency of one test to assign more positive test results than the other^23,24^. The degree of agreement between tests was interpreted as following: ≤ 0 = poor, 0.01–0.2 = slight, 0.21–0.4 = fair, 0.41–0.6 = moderate, 0.61–-0.8 = substantial, 0.81–1 = almost perfect^25^.

## Results

### Prevalence and risk factors

*T. evansi* infection was tested in 370 dromedary camels using TBS, CATT, and PCR tests. The percentage of camels that tested positive varied according on the diagnostic test, ranging from 17.3% (TBS) to 22.7% (PCR). There is a significant disparity in percentage assessed by TBS and CATT (*P* = 0.014), TBS and PCR (*P* = 0.000), and CATT and PCR (*P* = 0.002).

The prevalence of PCR-positive camels in associated risk factors are presented in Table 1. The univariable analysis indicated significant association between *T. evansi* infection and camel age, sex, BCS and sampling season. Table 2 lists the variables that were kept in the final multivariable model. The risk of *T. evansi* infection in camel aged > 6 to 10 years and > 10 years were eight and nine times higher, respectively, compared to younger camels (1–3 years-old). Female camels were 2.6 times more likely than male camels to be infected with *T. evansi*. Furthermore, camels with poor BCS and sampled on spring were 4 and 2.5 times more likely to be *T. evansi* infected compared to camels with good BCS and sampled on autumn, respectively.

**Table 1:** Prevalence of camel trypanosomiasis among associated risk factors.

Variable	No. of examined	No. of PCR positive	Prevalence	95% CI	*P*-value
**Governorates**					
Qalyubia	120	19	15.8	10.3–23.5	0.069
Kafr Elsheikh	125	30	24.0	17.3–32.3	
Matrouh	125	35	28.0	20.8–36.5	
**Age**					
1–3 years	100	7	7.0	3.4–14.0	0.001
> 3–6 years	110	15	13.6	8.4–21.4	
> 6–10 years	90	31	34.4	25.4–44.8	
> 10 years	70	31	44.3	33.1–56.1	
**Sex**					
Male	150	20	13.3	8.8–19.8	0.000
Female	220	64	29.1	23.5–35.5	
**Season**					
Autumn	85	14	16.5	10.0–25.9	0.001
Summer	115	33	28.7	21.2–37.6	
Winter	85	9	10.6	5.6–19.1	
Spring	85	28	32.9	23.8–43.6	
**Body condition**					
Good	120	11	9.2	5.1–15.8	0.000
Poor	250	73	29.2	23.9–35.2	
Total	370	84	22.7	18.7–27.3	

*CI* confidence interval.

**Table 2:** Multivariable logistic regression analysis of risk factors associated with *T. evansi* infection in dromedary camels.

Variables	OR (95% CI)	*P*-value
**Age**		
1–3 years	1.00 (ref.)	0.000
> 3–6 years	2.1 (0.8–5.5)	0.141
> 6–10 years	8.4 (3.3–21.5	0.000
> 10 years	9.0 (3.5–23.1)	0.000
**Sex**		
Male	1.00 (ref.)	
Female	2.6 (1.4–4.8)	0.002
**Season**		
Autumn	1.00 (ref.)	0.018
Summer	1.9 (0.9–4.2)	0.114
Winter	0.7 (0.3–1.8)	0.454
Spring	2.5 (1.1–5.7)	0.032
**Body condition**		
Good	1.00 (ref.)	
Poor	4.0 (1.9–8.4)	0.000

*OR* odds ratio, *CI* confidence interval.

### Diagnostic test characteristics

The test characteristics of TBS and CATT for detection of *T. evansi* infection in camels were evaluated against PCR results (Table 3). The CATT showed higher Se (0.83; 95% CI: 0.74–0.91) than TBS (0.76; 95% CI: 0.66–0.85). However, both tests showed perfect Sp (1.00). Furthermore, both tests had a high accuracy (≥ 95%) for detection of *T. evansi* infection in camels.

**Table 3:** Diagnostic test characteristics for thin blood smear (TBS) and card agglutination test for *T. evansi* (CATT) in 370 dromedary camels using PCR as the reference test.

Test characteristics^a^	TBS	CATT
True positives	64	70
False positives	0	0
True negatives	286	286
False negatives	20	14
Sensitivity (Se)	0.76 (0.66–0.85)	0.83 (0.74–0.91)
Specificity (Sp)	1.00 (0.99–1.00)	1.00 (0.99–1.00)
Positive predictive value (PPV)	1.00 (0.94–1.00)	1.00 (0.95–1.00)
Negative predictive value (NPV)	0.94 (0.90–0.96)	0.95 (0.92–0.97)
Accuracy	0.95	0.96

^a^Numbers in parentheses are 95% confidence intervals.

### Agreement between diagnostic tests

The kappa and PABAK estimates of agreement between diagnostic tests are presented in Table 4. The kappa values showed almost perfect agreement between tests. The PABAK estimates were numerically greater than the kappa values. However, the kappa (0.95; 95% CI: 0.90–0.99) and PABAK (0.97; 95% CI: 0.94–0.99) estimates of agreement between TBS and CATT were the highest.

**Table 4:** Cohen’s kappa (lower) and PABAK (upper) estimates, and 95% confidence interval for the agreement between different tests for detecting *T. evansi* in 370 dromedary camels.

Test	TBS	CATT	PCR
TBS	1.0	0.97 (0.94–0.99)	0.89 (0.85–0.94)
CATT	0.95 (0.90–0.99)	1.0	0.92 (0.89–0.96)
PCR	0.83 (0.76–0.90)	0.89 (0.83–0.94)	1.0

*TBS* thin blood smear, *CATT* card agglutination test for *T. evansi.*

## Discussion

Camels play an important role in Egyptian farmers' lives and livelihoods, both as draught animals and as a source of protein. However, a disease like surra has a negative impact on productivity with economic consequences for farmers. The goal of this study was to determine the prevalence of camel trypanosomiasis in three Egyptian governorates, using different diagnostic approaches such as parasite detection, serology, and molecular diagnostics and to assess their agreement.

In the present study, the prevalence of *T. evansi* infection in camels was 17.3% with TBS, which comparable to the estimated prevalence (12%) by the same test in Egypt^26^ and higher than 4.5% reported in camels from Ethiopia^27^ and the 0.7% reported in camels from Pakistan^12^. On the other hand, the CATT technique demonstrated that *T. evansi* infection was found in 18.9% of the investigated camels in the present study. This finding is lower than the 43.5% and 47.7% reported previously in camels from Egypt^28^ and Pakistan^12^, respectively. The PCR technique showed the highest proportion (22.7%) of camels infected with *T. evansi*, which is consistent with the rates (20.2% and 21.8%) reported in recent studies from Egypt^15,29^ and higher than the previously reported rates 4.1%^30^ and 3.8%^31^. Furthermore, the PCR estimated prevalence in the present study was higher than 11.2% reported in camels from South Algeria^5^, but lower than the 30%^32^ and 31.9%^12^ reported in camels from Palestine and Pakistan, respectively. The variation in the *T. evansi* prevalence could be attributed to sample size, sampling technique and vector density in the study area^33^. The significant difference in proportion of positivity determined by each test is expected and is consistent with previous study in camels from Kenya^34^. Several factors could explain the variation in proportions of *T. evansi* positivity detected by each test including the low Se of TBS which had a lower detection limit of 10^5^ trypanosomes/mL^3,30,35^. In contrast, PCR has the capacity to identify and amplify low quantities of parasite DNA in the bloodstream^34,36^. Furthermore, chronic infections may remain false negative with parasitological (TBS) and PCR examinations; however, successful treatment may result in serological (CATT) false positive as antibodies persist in circulation for several months^37,38^.

In the present study, the risk of *T. evansi* infection increased with the camel age, with camels older than 6 years having the highest risk of infection than young camels (1–3 years). Similarly, previous reports found that *T. evansi* infection or seropositivity were higher on adult camels (> 4 years) than young ones^5,13,39^. However, other studies have found a higher rate of *T. evansi* infection in young camels^33,40^ and no association between of *T. evansi* infection and camel age^41,42^. The high risk of *T. evansi* infection in older camels could be attributed to the chronic nature of the disease and intermittent parasitaemia, poor management and stress associated with the use of camels in draught. Furthermore, adult camels pasturing for long distances making them more vulnerable to the vector^12,43,44^.

Females are three times more likely than males to become infected, according to the current study. This finding is consistent with previous studies that found females to be at a higher risk of infection than males^5,27^, which was attributed to lactation and pregnancy, that weaken resistance and make them more susceptible to infection. However, another study found that males are more susceptible to infection than females due to physical work-related stress and exhaustion, movement in search of food and water, and thus increased vector exposure^45^. Furthermore, few studies have reported no differences in *T. evansi* seroprevalence between males and females^5,39^.

Season has a direct influence on the spreading of biting flies, which are responsible for the mechanical transmission of *T. evansi*^46^. The risk of *T. evansi* infection in this study was significantly higher in spring than autumn, which consistent with the study of Sobhy, et al.^13^, they found that spring, followed by summer, was the most favourable season for *T. evansi* infection in Egyptian camels.. This finding is not surprising given that several studies have linked a higher risk of *T. evansi* infection to dry seasons^3,47^, because of the favorable environmental conditions for fly vegetation and thus increased vector density^14,48^.

In this study, camels with poor body condition had a higher risk of *T. evansi* infection compared to camels with good body condition. Similar results have been reported in camels from Nigeria^8^, which contrasts with the findings of Gerem, et al.^27^, who found non-significant association between *T. evansi* infection and camel body condition in Ethiopia. There is a link between animal immunity and level of nutrition was reported^49^, thus camels with poor body condition are unable to resist infection.

The Se and Sp of TBS and CATT were assessed against PCR. The Se of TBS was higher than the 27.02% reported in camels^36^. However, the Se of CATT was comparable to the 86.9% and 78% reported in Mauritania^50^ and Indonesia^51^, respectively and higher than the 68.6% reported in camels from Kenya^52^. Both the TBS and CATT showed high Sp (100%). Similar results have been reported in camels from Indonesia^51^ and Kenya^52^, but lower Sp (83.03%) was reported in camels from Mauritania^50^. The level of agreement between the three tests was almost perfect (κ ≥ 0.83), which higher than the 0.3 reported between PCR and CATT^34^ and the poor agreement reported between TBS and both PCR and CATT^12^.

## Conclusions

Results of the present study showed a high rate of *T. evansi* infection in camels in three Egyptian governorates and risk factors associated with the infection. The CATT is a good candidate for routine use in the control program of trypanosomiasis in Egypt based on its Se and Sp and the fact that is easy to perform.

## Data Availability

All data generated or analysed during this study are included in this published article.
